# High-flow nasal oxygen vs. standard oxygen therapy in immunocompromised patients with acute respiratory failure: study protocol for a randomized controlled trial

**DOI:** 10.1186/s13063-018-2492-z

**Published:** 2018-03-05

**Authors:** Elie Azoulay, Virginie Lemiale, Djamel Mokart, Saad Nseir, Laurent Argaud, Frédéric Pène, Loay Kontar, Fabrice Bruneel, Kada Klouche, François Barbier, Jean Reignier, Anabelle Stoclin, Guillaume Louis, Jean-Michel Constantin, Julien Mayaux, Florent Wallet, Achille Kouatchet, Vincent Peigne, Pierre Perez, Christophe Girault, Samir Jaber, Johanna Oziel, Martine Nyunga, Nicolas Terzi, Lila Bouadma, Christine Lebert, Alexandre Lautrette, Naike Bigé, Jean-Herlé Raphalen, Laurent Papazian, Antoine Rabbat, Michael Darmon, Sylvie Chevret, Alexandre Demoule

**Affiliations:** 1 Medical Intensive Care Unit, APHP, Hôpital Saint-Louis. ECSTRA Team, and Clinical Epidemiology, UMR 1153, (Center of Epidemiology and Biostatistics, Sorbonne Paris Cité, CRESS), INSERM, Paris Diderot Sorbonne University, Paris, France; 2 Intensive Care Unit, Paoli Calmettes Institut, Marseille, France; 3 Critical Care Center, CHU de Lille, Lille, France; 4 Medical Intensive Care Unit, Hospices Civils de Lyon, Hôpital Edouard Herriot, Lyon, France; 5 Medical Intensive Care Unit, Hôpital Cochin, APHP, Université Paris Descartes, Paris, France; 6 Medical Intensive Care Unit and INSERM U1088, Amiens University Hospital, Amiens, France; 7 Medical Intensive Care Unit, André Mignot Hospital, Versailles, France; 8 Medical Intensive Care Unit, CHU de Montpellier, Montpellier, France; 9 Medical Intensive Care Unit, La Source Hospital, CHR Orléans, Orléans, France; 10 Medical Intensive Care Unit, Hotel Dieu, CHU de Nantes, Nantes, France; 11 Intensive Care Unit, Institut Gustave Roussy, Villejuif, France; 12 Intensive Care Unit, CHR de Metz-Thionville, Metz, France; 13 Department of Perioperative Medicine, CHU Clermont-Ferrand, Clermont-Ferrand, France; 14 Medical Intensive Care Unit and Respiratory Division, La Pitié-Salpêtrière University Hospital; Neurophysiologie Respiratoire Expérimentale et Clinique, Sorbonne Universités, UPMC Univiversité Paris 06, INSERM, UMRS_1158, Paris, France; 15 Intensive Care Unit, Lyon Sud Medical Center, Lyon, France; 16 Medical Intensive Care Unit, CHRU, Angers, France; 17 Intensive Care Unit, Centre Hospitalier Métropole-Savoie, Chambery, France; 18 Medical Intensive Care Unit, Hôpital Brabois, Vandoeuvre Les Nancy, France; 19 Medical Intensive Care Unit, Hôpital Charles Nicolle, Rouen, France; 20 Department of Anesthesiology and Critical Care Medicine B (DAR B), Saint-Eloi Hospital, University Teaching Hospital of Montpellier; INSERM U1046, CNRS, UMR 9214, Montpellier, France; 21 Medical Intensive Care Unit, Avicenne University Hospital, Bobigny, France; 22 Intensive Care Unit, Roubaix Hospital, Roubaix, France; 23 Medical Intensive Care Unit, CHU de Grenoble Alpes, Grenoble, France; 24 Medical Intensive Care Unit, CHU Bichat, Paris, France; 25 Intensive Care Unit, Centre Hospitalier Départemental Les Oudairies, La Roche Sur Yon, France; 26 Medical Intensive Care Unit, Gabriel-Montpied University Hospital, Clermont-Ferrand, France; 27 Medical Intensive Care Unit, CHU Saint-Antoine, Paris, France; 28 Department of Anesthesia and Critical Care, Necker Hospital, Paris, France; 29 Réanimation des Détresses Respiratoires et Infections Sévères, Assistance Publique – Hôpitaux de Marseille, Hôpital Nord, Aix-Marseille Université, Faculté de Médecine, Marseille, France; 30 Respiratory Intensive Care Unit, Hôpital Cochin, Paris, France; 31 Medical Intensive Care Unit, Hôpital Nord, Saint Etienne, France; 32 Biostatistics department, Saint Louis Teaching Hospital, Paris, France

**Keywords:** Acute respiratory failure, Immunosuppression, Immunocompromised Hematology, Mortality, High-flow oxygen, Oxygen, Intubation

## Abstract

**Background:**

Acute respiratory failure (ARF) is the leading reason for intensive care unit (ICU) admission in immunocompromised patients. High-flow nasal oxygen (HFNO) therapy is an alternative to standard oxygen. By providing warmed and humidified gas, HFNO allows the delivery of higher flow rates via nasal cannula devices, with FiO_2_ values of nearly 100%. Benefits include alleviation of dyspnea and discomfort, decreased respiratory distress and decreased mortality in unselected patients with acute hypoxemic respiratory failure. However, in preliminary reports, HFNO benefits are controversial in immunocompromised patients in whom it has never been properly evaluated.

**Methods/design:**

This is a multicenter, open-label, randomized controlled superiority trial in 30 intensive care units, part of the Groupe de Recherche Respiratoire en Réanimation Onco-Hématologique (GRRR-OH). Inclusion criteria will be: (1) adults, (2) known immunosuppression, (3) ARF, (4) oxygen therapy ≥ 6 L/min, (5) written informed consent from patient or proxy. Exclusion criteria will be: (1) imminent death (moribund patient), (2) no informed consent, (3) hypercapnia (PaCO_2_ ≥ 50 mmHg), (4) isolated cardiogenic pulmonary edema, (5) pregnancy or breastfeeding, (6) anatomical factors precluding insertion of a nasal cannula, (7) no coverage by the French statutory healthcare insurance system, and (8) post-surgical setting from day 1 to day 6 (patients with ARF occurring after day 6 of surgery can be included).

The primary outcome measure is day-28 mortality. Secondary outcomes are intubation rate, comfort, dyspnea, respiratory rate, oxygenation, ICU length of stay, and ICU-acquired infections.

Based on an expected 30% mortality rate in the standard oxygen group, and 20% in the HFNO group, error rate set at 5%, and a statistical power at 90%, 389 patients are required in each treatment group (778 patients overall). Recruitment period is estimated at 30 months, with 28 days of additional follow-up for the last included patient.

**Discussion:**

The HIGH study will be the largest multicenter, randomized controlled trial seeking to demonstrate that survival benefits from HFNO reported in unselected patients also apply to a large immunocompromised population.

**Trial registration:**

ClinicalTrials.gov, ID: NCT02739451. Registered on 15 April 2016.

**Electronic supplementary material:**

The online version of this article (10.1186/s13063-018-2492-z) contains supplementary material, which is available to authorized users.

## Background

Acute respiratory failure (ARF) is the leading reason for intensive care unit (ICU) admission of immunocompromised patients [1–6]. Mortality has decreased dramatically in this population in recent years, for several reasons. Management strategies for the underlying conditions have benefited from a number of innovations such as steroid-sparing agents, watch-and-wait approaches, and targeted therapies [7, 8]. Early ICU admission to permit the use of non-invasive diagnostic and therapeutic strategies has increased survival [1, 9–11]. Finally, the introduction of other oxygenation strategies has improved the management of respiratory dysfunction (Table 1).

**Table 1:** Definitions for oxygen delivery devices and reported outcomes using high-flow nasal oxygen (HFNO)

Definitions	
HFNO	Device that delivers humidified and warmed, high-flow oxygen at flows greater than 15 L/min
Usual oxygen therapy devices	Devices used to treat spontaneously ventilating patients in the intensive care unit (ICU) who require supplemental oxygen. They deliver either: low-flow oxygen (including nasal cannula, Ventimask® without Venturi effect, and non-rebreather mask) or medium-flow oxygen (Venturi masks and medium-flow facemasks)
Non-invasive ventilation (NIV)	Administration of ventilatory support without using an endotracheal tube or tracheostomy tube. Ventilatory support can be provided through diverse interfaces (mouthpiece, nasal mask, facemask, or helmet) using a variety of ventilatory modes (e.g., volume ventilation, pressure support, bi-level positive airway pressure (BiPAP; see the image below), proportional-assist ventilation (PAV), and continuous positive airway pressure (CPAP)) with either dedicated NIV ventilators or ventilators also capable of providing support through an endotracheal tube or mask
Clinical outcomes in HFNO	Assessed by measuring
Oxygenation (desaturation)	Continuous SpO_2_ PaO_2_ at fixed times PaO_2_/FiO_2_ ratio
Ventilation	PaCO_2_
Airway pressures	Nasopharyngeal or hypopharyngeal catheter
Work of breathing	Respiratory rate
Patient comfort and adherence	Visual Analog Scale (VAS) for breathing difficulties Satisfaction and tolerance; global comfort Dyspnea (VAS or Borg scale); dry mouth
Cardiovascular status	Heart rate Shock; need for vasopressors
Complications	Need for NIV Need for intubation and mechanical ventilation (MV); mortality

Oxygen therapy is the first-line treatment in hypoxemic patients. Oxygen can be delivered using low-flow devices (up to 15 L/min) such as nasal cannulas, non-rebreathing masks, and bag-valve masks. The fraction of inspired oxygen (FiO_2_) obtained using these devices varies with the patient’s breathing pattern, peak inspiratory flow rate, delivery system, and mask characteristics. Maximum flow rates are limited in part by the inability of these devices to heat and humidify gas at high flows. Also, if the patient has a high inspiratory flow rate, the amount of entrained room air is large and dilutes the oxygen, thereby lowering the FiO_2_.

Over the past two decades, devices that deliver heated and humidified oxygen at high flow rates through a nasal cannula were developed as an alternative to low/medium-flow devices. High-flow nasal oxygen (HFNO) delivers oxygen flow rates of up to 60 L/min. An air/oxygen blender is connected via an active heated humidifier to a nasal cannula and allows FiO_2_ adjustment independently from the flow rate. Compared to other devices, HFNO provides a number of physiological benefits including greater comfort and tolerance, more effective oxygenation under some circumstances and breathing pattern improvements with an increase in tidal volume and decreases in respiratory rate and dyspnea (Tables 2 and 3). These benefits are broadening the indications of HFNO, which has now been evaluated and used to treat hypoxemic respiratory failure, to improve oxygenation for pre-intubation, and to treat patients after surgery or after extubation (Table 4). Moreover, recent high-quality randomized controlled trials (RCTs) have confirmed previous preliminary results [12–14]. Nevertheless, controlled studies in specific patient populations, such as immunocompromised patients, are needed to confirm that HFNO is clinically superior over other methods, to evaluate effects on survival, and to determine the optimal indications of HFNO compared to other modalities such as standard oxygen therapy and non-invasive ventilation (NIV).

**Table 2:** Drawbacks of standard oxygen therapy that limit the effectiveness and tolerance of oxygen delivery [15–21]

Oxygen is not humidified at low flow:
dry nose dry throat dry mouth nasal pain ocular irritation, nasal and ocular trauma discomfort related to the mask gastric distension aspiration global discomfort
Insufficient heating leads to poor tolerance of oxygen therapy
Unwarmed and dry gas may cause bronchoconstriction and may decrease pulmonary compliance and conductance
With low/medium-flow devices, oxygen cannot be delivered at flows greater than 15 L/min, whereas inspiratory flow in patients with respiratory failure varies widely and is considerably higher, between 30 and more than 100 L/min
Given the difference between the patient’s inspiratory flow and the delivered flow, FiO_2_ is both variable and often lower than needed

**Table 3:** Physiological benefits of high-flow nasal oxygen (HFNO) compared to conventional oxygen therapy [24–41]

*FiO*_*2*_ *values are higher and more stable*
because the delivered flow rate is higher than the spontaneous inspiratory demand and because the difference between the delivered flow rate and the patient’s inspiratory flow rate is smaller. ☞ The flow rate must be set to match the patient’s inspiratory demand and/or the severity of the respiratory distress
*The anatomical dead space is decreased, via washout of the nasopharyngeal space*
Consequently, a larger fraction of the minute ventilation reaches the alveoli, where it can participate in gas exchange. Respiratory efforts become more efficient. Thoraco-abdominal synchrony improves
*The work of breathing is decreased*
because HFNO mechanically stents the airway, provides flow rates that match the patient’s inspiratory flow, and markedly attenuates the inspiratory resistance associated with the nasopharynx, thereby eliminating the attendant work of breathing
*The gas delivered is heated and humidified*
Warm humid gas reduces the work of breathing and improves muco-ciliary function, thereby facilitating secretion clearance, decreasing the risk of atelectasis, and improving the ventilation/perfusion ratio and oxygenation. The body is spared the energy cost of warming and humidifying the inspired gas. Warm humid gas is associated with better conductance and pulmonary compliance compared to dry, cooler gas. ☞ HFNO delivers adequately warmed and humidified gas only when the flow rate is > 40 L/min
*Positive airway pressures are increased*
The nasal cannula generates continuous positive pressures in the pharynx of up to 8 cmH_2_O. The positive pressure distends the lungs, ensuring lung recruitment and decreasing the ventilation-perfusion mismatch in the lungs. End-expiratory lung volume is greater with HFNO than with low-flow oxygen therapy. ☞ Minimising leaks around the cannula prongs is of the utmost importance

**Table 4:** Clinical studies on high-flow nasal oxygen (HFNO) therapy in adults with hypoxemic acute respiratory failure (ARF) [44–46]

Reference	Study design	Population	*N* patients	Results
Hypoxemic acute respiratory failure in the ICU				
[22]	Cohort, unselected patients. HFNO 50 L/min vs. face-mask oxygen	Hypoxemic ARF	38	Improved oxygenation Decreased respiratory rate
[23]	Cohort, unselected patients. HFNO 20–30 L/min vs. face-mask oxygen	Hypoxemic ARF	20	Improved oxygenation Decreases in respiratory/heart rates, dyspnea, respiratory distress, and thoraco-abdominal asynchrony
[47]	HFNO compared to face-mask oxygen	Hypoxemic ARF	60	Decreased treatment failure (defined as need for NIV) from 30% to 10%. Fewer desaturation episodes
[48]	Cohort study. HFNO 20–30 L/min vs. face-mask oxygen	Hypoxemic ARF	20	Improved comfort; improved oxygenation
[49]	Cohort study (post hoc)	Hypoxemic ARF (2009 A/H1N1v outbreak)	20	9/20 (45%) success (no intubation). All 8 patients on vasopressors required intubation within 24 h. After 6 h of HFNO, non-responders had lower PaO_2_/FiO_2_ values and needed higher oxygen flow rates.
[43]	Observational, single-centre study	ARDS	45	40% intubation rate. HFNO failure associated with higher SAPSII, development of additional organ failure, and trends toward lower PaO_2_/FiO_2_ values and higher respiratory rates
[13]	Multicentre, open-label RCT with 3 groups. HFNO, usual oxygen therapy (face mask), or non-invasive positive-pressure ventilation	Hypoxemic ARF, PaO_2_/FiO_2_ ≤ 300	310	Intubation rate was 38% with HFNO, 47% with standard oxygen, and 50% with NIV. The number of ventilator-free days by day 28 was significantly higher with HFNO. Decreased day-90 mortality with HFNO
[50]	Retrospective before/after study of HFNO	Hypoxemic ARF	172	Reduced need for ventilation (100% vs 63%, *p* < 0.01) and decreased ventilator-free days
[42]	Patients intubated after HFNO	Hypoxemic ARF	175	In patients intubated early, lower mortality (39.2 vs. 66.7%), higher extubation success (37.7% vs. 15.6%) and more ventilator-free days. Early intubation was associated with decreased ICU mortality
Hypoxemic acute respiratory failure in the ED				
[51]	Patients with ARF (> 9 L/min oxygen or clinical signs of respiratory distress)	Hypoxemic ARF	17	Decreased dyspnea and respiratory rate and improved oxygenation
[52]	RCT of HFNO vs. standard oxygen for 1 h	Hypoxemic ARF	40	Decreased dyspnea and improved comfort

*ARDS* acute respiratory distress syndrome, *ICU* intensive care unit, *NIV* non-invasive ventilation, *RCT* randomized controlled trial

Among patients with ARF, those with immunosuppression have higher mortality rates compared to unselected patients. The use of endotracheal mechanical ventilation is associated with higher mortality in immunocompromised patients. Therefore, management techniques that decrease the need for intubation may hold promise for decreasing mortality [53–56].

Four studies evaluated the feasibility and safety of HFNO in immunocompromised patients with ARF. In a retrospective, single-center study reported in 2013, the feasibility of HFNO was evaluated in 45 patients with hematological malignancies [57]. Of the 45 patients, 15 recovered without intubation (33%); their hospital mortality rate was 2/15 (13%), compared to 26/30 (87%) of the patients who failed HFNO and required intubation. HFNO failure was significantly associated with bacterial pneumonia as the cause of ARF. In a single-centre study of patients with solid tumors reported in 2011, of 183 patients taken at random from the institutional database, 132 (72%) had received HFNO in the ICU to treat hypoxia [58]. Among them, 41% improved and 44% remained stable while on HFNO, whereas 15% declined. A 2013 report describes a study in 30 patients with advanced cancer and persistent dyspnea that used a randomized design to compare the physiological effects of HFNO vs. bi-level positive airway pressure (BiPAP) for 2 h [59]. Both treatments similarly improved the dyspnea, as assessed using a Visual Analog Scale (VAS) and the modified Borg scale, and non-significantly diminished the respiratory rate. Oxygen saturation improved only with HFNO. Neither technique induced major adverse effects. The last study, published in 2015, evaluated HFNO for treating ARF requiring ICU admission in 37 lung transplant recipients [60]. HFNO proved feasible and safe and decreased the absolute risk of intubation by 29%, with a number-needed-to-treat to avoid one intubation of three. Last, in a study of 50 Do-Not-Intubate patients with hypoxemic respiratory distress, including a third of immunocompromised patients, HFNO allowed an improvement in oxygenation and decreased respiratory rate [61].

Four studies assessed HFNO efficacy in immunocompromised patients with ARF. The first study, by Mokart et al., analyzed a retrospective cohort of 178 patients with cancer and ARF (O_2_ > 9 L/min), including 76 (43%) treated with NIV + HFNO, 74 (42%) with NIV + low/medium-flow O_2_, 20 (11%) with HFNO alone, and 8 with low/medium-flow O_2_ alone [62]. NIV + HFNO was associated with lower mortality (37% vs. 52% in remaining patients, *p* = 0.04) and was independently associated with lower day-28 survival in a propensity-score analysis. Second, in a substudy of data from our recent iVNIctus RCT of early NIV in immunocompromised patients with ARF [63–65], 141/374 (38%) patients received HFNO, and either NIV or low/medium-flow oxygen was used in the other patients. To allow accurate adjustment, we built a propensity score using variables available at ICU admission. Intubation rate and day-28 mortality were not significantly different in the HFNO arm compared to the NIV or low/medium-flow oxygen arm. Third, in 115 immunocompromised patients with ARF, 60 (52%) were treated with HFN0 alone and 55 (48%) with NIV as first-line therapy with 30 patients (55%) receiving HFNO and 25 patients (45%) standard oxygen between NIV sessions [66]. The rates of intubation and 28-day mortality were higher in patients treated with NIV than with HFNO (55 vs. 35%, *p* = 0.04, and 40 vs. 20%, *p* = 0.02, respectively). Using propensity score-matched analysis, NIV was associated with mortality. Using multivariate analysis, NIV was independently associated with intubation and mortality. Last, in a post-hoc analysis of the FLORALI study that only included immunocompromised patients, 8 (31%) of 26 HFNO patients, 13 (43%) of 30 patients treated with standard oxygen, and 17 (65%) of 26 patients treated with NIV required intubation at 28 days (*p* = 0·04). Odds ratios for intubation did not differ, however, between HFNO patients and those receiving standard oxygen only [67]. Last, in the Efraim study that included 1611 immunocompromized patients with acute respiratory failure, the use of HFNO had an effect on intubation rate but not on mortality, whereas, failure to identify ARF etiology was associated with increased intubation rate and mortality [68].

Although the effects of HFNO have varied across studies, the data establish that this treatment modality is feasible and safe in immunocompromised patients. They also demonstrate that outcomes with HFNO are at least as good as with other oxygen therapy methods in this population. Thus, they warrant further trials to determine whether HFNO improves survival in unselected immunocompromised patients with hypoxemic ARF. Immunocompromised patients have specific treatment needs, as shown by their two-fold higher mortality rate after intubation compared to other patients. Data on HFNO in immunocompromised patients are conflicting.

We therefore designed the present RCT (HIGH). This RCT is a superiority study of HFNO vs. other oxygenation strategies (low/medium-flow oxygen) in immunocompromised patients requiring oxygen. The primary endpoint is day-28 survival. The patients will be recruited at 31 centers belonging to the Groupe de Recherche Respiratoire en Réanimation Onco-Hématologique (GRRR-OH), a research network that specializes in the management of critically ill immunocompromised patients and has a particularly high level of expertise in respiratory care strategies. The control group will receive low/medium-flow oxygen as deemed appropriate by the physician since the recent large iVNIctus trial by our group did not show any superiority of NIV on intubation rates or survival. The experimental group will receive continuous HFNO at any time after ICU admission, for pre-oxygenation before intubation, after extubation, and for any ICU procedure that might induce hypoxemia). HFNO will not be used in the control group.

## Methods/design

### Design and settings

**Fig. 1:**
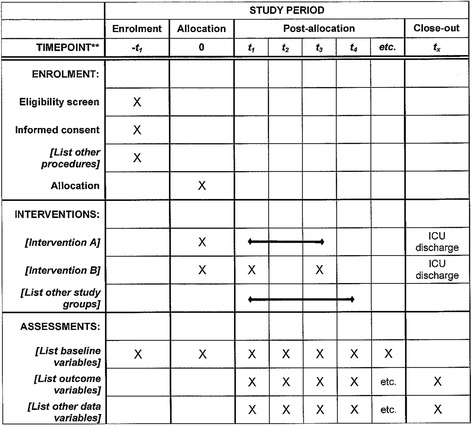
SPIRIT checklist

The HIGH trial is a prospective, multicenter, open-label RCT comparing HFNO vs. other oxygenation strategies (low/medium-flow oxygen) in immunocompromised patients requiring oxygen. The study hypothesis is that early HFNO decreases mortality on day 28 after randomization in immunocompromised patients requiring ICU admission for ARF. Figure 1 shows an adapted version of the SPIRIT Figure for the trial (Additional file 1).

### Ethical aspects

The study was approved by the local Independent Ethic Committee (Comité de Protection des Personnes CPP Ile de France IV, Saint Louis on 28 March 2016, number 2016/08), the French health authorities (AFSSAPS) on 14 March 2016, number EudraCT 2016-A00220-51. The University Hospital of Paris (AP-HP) and, by delegation, the Clinical Research and Development Department (DRCD) is the sponsor of the trial (sponsor code: P150912/IDRCB No: 2016-A00220-51). Informed consent will be obtained from each participant.

### Participating intensive care units

All participating centers belong to the GRRR-OH. All these centers have previously taken part in observational studies, surveys, or therapeutic trials. They all have high case-volumes of patients with immune deficiencies due to immunosuppressive drugs, solid-organ transplantation, malignancies, or systemic diseases. Although they are specialized in oncology and hematology, they also admit high volumes of patients with systemic diseases, solid-organ transplants and other immunosuppression.

### Study population

Eligible patients are immunocompromised patients who are admitted to the ICU and need oxygen supplementation (of at least 6 L/min) at any stage of their ICU stay. All randomized patients will be included in the full set of analysis (intent-to-treat basis).

To be randomized patients should fulfill all the following inclusion criteria (1) adult (age ≥ 18 years), (2) known immunosuppression defined as one or more of the following: immunosuppressive drugs/long-term (> 3 months) or high-dose (> 0.5 mg/kg/day) steroids, solid-organ transplant, solid tumor having required cancer care in the last 5 years, hematological malignancy or primary immune deficiency, (3) ICU admission for ARF, (4) need for oxygen therapy ≥ 6 L/min, and (5) written informed consent from the patient or proxy (if present) before inclusion or once possible when patient has been included in a context of emergency.

Exclusion criteria were: (1) imminent death (moribund patients), (2) refusal of study participation or to pursue the study by the patient, (3) hypercapnia with a formal indication for NIV (PaCO_2_ ≥ 50 mmHg, formal indication for NIV), (4) isolated cardiogenic pulmonary edema (formal indication for NIV). Patients with pulmonary edema associated with another ARF etiology can be included, (5) pregnancy or breastfeeding, (6) anatomical factors precluding the use of a nasal cannula, (7) absence of coverage by the French statutory healthcare insurance system, and (8) post-surgical setting from day 1 to day 6 (patients with ARF occurring after day 6 of surgery can be included).

### Randomization

Randomization will be stratified on three factors, measured at study inclusion, namely: (1) time since ICU admission, segregating day 0 (calendar date of ICU admission), day 1, day 2 vs. ≥ day 3; (2) hypoxemia severity, segregating oxygen flow < vs. ≥ 9 L to reach SpO_2_ ≥ 95% at randomization, and (3) shock, based on the administration of catecholamine. Thus, analysis could consider treatment-by-subset interaction on such strata.

Randomization will be achieved using an electronic system incorporated in the eCRF and R software (http://www.R-project.org/). The impact of the intervention will be assessed at the patient level. The randomization unit is the center. Randomization will be centralized on a web site to ensure allocation concealment at the trial statistical center. Patients will be randomized into two parallel groups in a 1:1 ratio. Randomization will be stratified (see above), resulting in eight different randomization lists that will be pre-specified and balanced through the use of permutation blocks of fixed size that will not be disclosed to the local investigators, to ensure allocation concealment and to avoid all risk of bias in patient selection.

### Study interventions

This open RCT will compare two oxygenation strategies.

#### Standard oxygen as the usual care (control group)

Patients in the control group will receive the best standard of care, according to the usual practice of the local intensivists and primary-care physicians. Oxygen therapy will be delivered using any device or combination of devices that are part of usual care: nasal oxygen, and mask with or without a reservoir bag and with or without the Venturi system. Oxygen settings are set to target a SpO_2_ ≥ 95%. HFNO will not be used in the control group. NIV will not be used at all in this trial, unless patients develop hypercapnia or pulmonary edema throughout the ICU stay, for the time they meet these conditions. ICU discharge will be allowed when patients will meet the ability to maintain SpO_2_ ≥ 95% with less than 6 L/min oxygen.

#### High-flow nasal oxygen (intervention group)

Patients in the HFNO group will receive the best standard of care, according to the usual practice of the local intensivists and primary physicians, with one exception: supplemental oxygen will be provided only by continuous HFNO. HFNO will be initiated at a flow rate of 50 L/min and 100% FiO_2_. If the target SpO_2_ is not reached, the flow rate will be increased to 60 L/min. Then, FiO_2_ will be tapered to target a SpO_2_ ≥ 95%. The minimal flow rate within the first 3 days will be 50 L/min. In patients who require intubation, HFNO will be used during laryngoscopy and immediately after extubation. Also, HFNO will be used before, during, and after all ICU procedures. Patients with discomfort due to HFNO will have their flow rate decreased until the discomfort resolves. If the nasal prongs generate significant discomfort or skin breakdown, a Venturi mask will be used instead until HFNO can be used again; except in this situation, standard oxygen will be used in the intervention group. NIV will, however, be used in the same conditions than in the control group.

HFNO will be stopped based on clinical criteria (improvement of clinical signs of respiratory distress), PaO_2_/FiO_2_ > 300, and ability to maintain SpO_2_ ≥ 95% with less than 6 L/min of standard oxygen (allowing ICU discharge as HFNO may not be available in the wards).

### Data collection and follow-up

#### Evaluation at study inclusion (T0)

The evaluation at study inclusion will include patient’s characteristics, underlying disease, associated organ dysfunction, investigations usually performed at ICU admission in immunocompromised patients with ARF, and ARF etiology.

#### Evaluations throughout study participation

Evaluations performed throughout study participation will include physiological variables including respiratory and ventilation parameters (respiratory rate, SpO_2_, oxygen flow and/or FiO_2_), blood gases and chest x-ray (the worst values will be recorded). Results of investigations, ICU-acquired infections and data on oxygenation tolerance and efficacy as well as on comfort will be also collected.

ICU-acquired infections are defined as any new-onset infection starting more than 48 h after ICU admission for which the clinical team started a new antibiotic regimen. Every single infection diagnosis will be made using Centers for Disease Control and Prevention definitions [69].

#### Evaluation at the end of study participation

Evaluations performed at the end of study participation will consist of mortality on day 28, need for intubation, ICU and hospital lengths of stay and ICU-acquired infections. All elements allowing to record secondary endpoints will be collected.

### Organization of the trial

#### Funding and support

The HIGH trial is promoted by the Assistance Publique – Hôpitaux de Paris and supported by a grant from the French Ministry of Health (Programme Hospitalier de Recherche Clinique 2012; AOM12456).

#### Coordination and implementation of the trial

Each medical and paramedical team in the 31 participating ICUs were trained in the protocol and data collection using an electronic case-record form during formal meetings prior to screening and inclusion. The electronic case-record form was developed with CleanWEBTM, a centralized, secure, interactive, web-response system accessible from each study center, provided and managed by Telemedicine Technologies.

Local physicians and clinical research assistants in each participating ICU are responsible for daily screening and inclusion of patients, compliance with protocol, availability of data requested for the trial and completion of the electronic case-record form. In accordance with French law, the electronic case-record form and database were validated by the appropriate committees (Comité Consultatif sur le Traitement de l’Information en matière de Recherche dans le domaine de la Santé; Commission Nationale de l’Informatique et des Libertés).

#### Interim analysis

One interim analysis by an independent Data Safety and Monitoring Board is planned after the occurrence of 100 deaths. The Data Safety and Monitoring Board will be blinded to allocation of groups and may decide premature termination of the study. The board consists of one methodologist, one pulmonologist, and one intensivist. Data are blindly analyzed but unblinding is possible on request of the Data Safety and Monitoring Board. An extraordinary meeting may be requested by the principal investigator or the methodologist, in the case of unexpected events that might affect continuation of the protocol.

### Blinding

Given the nature of the interventions, physicians, nurses, and patients cannot be blinded for the randomized interventions. The analysis will be blinded to allocation of groups.

### Study outcomes

#### Primary endpoint

The primary endpoint of this trial is day-28 mortality.

#### Secondary endpoints

The secondary endpoints are: intubation rate (proportion of patients requiring invasive mechanical ventilation) on day 28, patient comfort VAS, dyspnea (VAS and Borg scale), respiratory rate, oxygenation (based on the lowest SpO_2_ value and on PaO_2_/FiO_2_ from day 1 to day 3, ICU stay length, incidence of ICU-acquired infections.

### Statistical methods

All statistical analyses will be performed using SAS (SAS Inc., Cary, NC, USA) and R (http://www.R-project.org/) software.

#### Sample size calculation

Based on a 30% day-28 mortality rate in usual-care oxygen group, and a 20% day-28 mortality rate in the HFNO group, with α set at 5%, to obtain a 90% power for demonstrating superiority for the primary outcome, we need 778 patients (389 in each group).

Recruitment is expected to take 30 months, and 28 additional days will be required for follow-up.

#### Interim analyses

One interim analysis will be performed once 100 deaths will have been observed. Due to inflation of type I error consideration, it will use the Haybittle-Peto boundary, that is a *p* value threshold of 0.001 for the interim analysis (while the terminal analysis will use a threshold of 0.05, as scheduled in the sample size computation). Moreover, to get insight in the difference across arms in terms of futility or efficacy, the Bayesian posterior probability of the 28-day mortality rate and of the log odds ratio will be computed, using a uniform non-informative prior. The final analysis will be started after inclusion of the planned number of patients.

#### Methodology of the statistical analysis

The main comparison based on the intention-to-treat principle will compare the intervention arm to the control arm on the full-set of randomized patients. The primary hypothesis is superiority of the NIV in terms of 28-day mortality (primary outcome). For all secondary outcomes, our hypothesis is that HFNO is superior over standard oxygen, with two-sided *p* values for comparison tests. Secondary and exploratory comparisons of the primary endpoint will look for treatment-by-covariate interactions according to the subsets defined above. Finally, a per-protocol analysis will be performed.

#### Missing values and outliers

Missing values for the main outcome measure are not expected to be observed; nevertheless, in case of occurrence, they will be handled using time-to-event methods in which each patient contributes to the estimate of failure time distribution until they are lost-to-follow up or withdrawn from the study using competing-risks estimates. Missing values for predictors will be imputed using multiple imputation techniques.

#### Analysis of the primary outcome

The main endpoint is binary, as all patients will be followed until day 28, at which time they will be classified as alive or dead. The relative risk of hospital death in the experimental vs. the control arm will be estimated to assess the effectiveness of the intervention, with 95% confidence interval. Analyses adjusted on potential confounders will be performed. Intervention-by-subsets interactions will be tested using Gail and Simon statistics. In case of significant interaction, subset analyses will be performed on each subset.

#### Analysis of the secondary outcomes

Competing-risk endpoints (ICU-acquired events including intubation, ICU-acquired infection) will be analyzed using competing-risk methods. Specifically, cumulative incidences of the event of interest will be estimated, taking into account the competition between death or discharge alive from the ICU and the event of interest, then compared using the Gray test. Adjustment for potential confounders will be based on cause-specific Cox models. ICU length of stay will be analyzed overall and in survivors and dead patients, separately. The former analysis will be based on Kaplan-Meier estimates while the later on the competing-risk estimator, as described above. Analyses of longitudinal outcomes (oxygenation, dyspnea, patient’s comfort) will be based on joint models, taking into account the right censoring of the data.

## Discussion

ARF remains the most frequent and challenging life-threatening event in patients with hematological malignancies. In patients with prolonged neutropenia (acute leukemia or bone marrow transplant recipients), respiratory events occur in up to half of cases, of which a further half are complicated by ARF. Despite a recent improvement in survival, intubation and subsequent invasive mechanical ventilation remains associated with high mortality in immunocompromised patients with ARF. In recent studies, mortality after intubation was 60% in hematological patients and 40% in immunocompromised patients. In that setting, any strategy that could prevent intubation and subsequent increase in mortality could be of benefit.

HFNO has been associated with an increase survival for immunocompetent patients managed in the ICU for a hypoxemic ARF, and with a decrease in intubation rate in the most hypoxemic patients. Nevertheless, data are scarce in specific patient populations, such as immunocompromised patients, who are at high risk of intubation when presenting with ARF. Clearly, data are needed to confirm that HFNO is clinically superior over other methods in immunocompromised patients. This fully justifies the HIGH trial.

As a consequence of the negative result of our recent iVNIctus multicentre RCT that did not show a benefit of NIV on mortality nor on intubation in immunocompromised patients with ARF, we have decided that NIV would not delivered in a systematic way to the patients included in the HIGH trial. In addition, recent data from an ancillary study of the FLORALI trial suggests that intubation rate and mortality were higher in patients treated with NIV than in those treated with HFNO. However, clinicians in charge will be allowed to deliver NIV to patients with a well-established indication of NIV, such as cardiogenic pulmonary edema and hypercapnic ARF.

We expect the HIGH trial to assess an oxygenation management strategy including HFNO. We hypothesize that mortality will be lower in patient receiving HFNO, possibly in association with a reduction of the intubation rate. We also expect the HIGH trial to analyze the factors that predict intubation in immunocompromised patients with ARF.

## Trial status

Enrollment is ongoing, having started on May 2016. The first interim analysis was conducted on 13 March 2017, and the Data Safety and Monitoring Board recommended that the study be continued. On 13 November 2017, 686 patients were included in the trial. Enrollment is expected to be completed in February 2018.

## Additional file

Additional file 1: SPIRIT 2013 Checklist: recommended items to address in a clinical trial protocol and related documents*. (PDF 142 kb)

## Abbreviations

ARF: Acute respiratory failure
GRRR-OH: Groupe de Recherche Respiratoire en Réanimation Oncohématologique
HFNO: High-flow nasal oxygen
ICU: Intensive care unit
NIV: Non-invasive ventilation
